# Endovascular management of portal steal syndrome due to portosystemic shunts after living donor liver transplantation

**DOI:** 10.1002/jgh3.12540

**Published:** 2021-04-04

**Authors:** Surabhi Jajodia, Anubhav H Khandelwal, Rohit Khandelwal, Abhay K Kapoor, Sanjay S Baijal

**Affiliations:** ^1^ Department of Clinical Imaging and Interventional Radiology Apollo Hospitals Kolkata India; ^2^ Department of Interventional Radiology Medanta – The Medicity, Gurugram Haryana India

**Keywords:** hepatic encephalopathy, living donor liver transplantation, portal steal, stent graft, vascular plugs

## Abstract

**Background and Aim:**

After liver transplant, pre‐existent porto‐systemic shunts (PSS) may persist, causing “portal steal,” leading to graft dysfunction, hepatic encephalopathy (HE), and eventual rejection. In recipients of small‐for‐size transplant liver grafts, shunts may be created intraoperatively, facilitating diversion of portal flow to systemic circulation to avoid ill‐effects of portal overperfusion. These iatrogenic shunts may also subsequently lead to portal steal. We aim to evaluate safety and efficacy of endovascular techniques in management of portal steal due to PSSs in living donor liver transplantation (LDLT) recipients.

**Methods:**

Between 2013 and 2020, we encountered five LDLT recipients with large PSS, who presented with graft dysfunction and/or HE. One patient had a surgically created shunt and four had spontaneous shunts, not surgically ligated during transplant. Endovascular techniques including plug‐assisted or balloon‐occluded retrograde transvenous obliteration (PARTO/BRTO) or covered inferior vena cava (IVC) stent grafts were to occlude these PSS and counter the portal steal in all patients. Technical success and clinical outcomes at 1‐year‐follow‐up were assessed.

**Results:**

Imaging showed large PSS causing portal steal syndrome in all five patients. IVC stent graft was used to isolate the shunt in two patients and PARTO/BARTO was performed in three patients. One patient had guarded prognosis due to multiple organ dysfunction and died 5 days after endovascular procedure. At 1‐year follow up, graft functions normalized in four patients with no recurrence of HE. No procedure‐related complications were seen.

**Conclusion:**

Endovascular techniques can be safely and effectively used to counter portal steal syndrome in LDLT recipients, thus avoiding surgical re‐exploration in these patients.

## Graphical abstract

Living donor liver transplantation (LDLT) is one of the most rapidly expanding organ transplant programs in the world. Portal steal is a rare but potentially correctable cause of graft dysfunction in LDLT recipients and traditionally is managed by surgical ligation. There are few published individual case reports where endovascular techniques, including balloon‐occluded retrograde transvenous obliteration, plug‐assisted retrograde transvenous obliteration, and inferior vena cava stenting, have been used as minimally invasive techniques to obliterate portosystemic shunts in LDLT recipients, thereby averting a surgical re‐exploration and associated complications. We present our experience of five LDLT recipients with portal steal where we successfully used endovascular techniques of management.

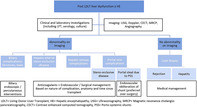

## Introduction

Portal hypertension is a known complication of cirrhosis. More than 40% of patients with end‐stage liver disease (ESLD) spontaneously develop portosystemic collaterals to compensate for increase in intravascular resistance.^1^ Living donor liver transplantation (LDLT) has been a useful therapeutic option for ESLD in the past few decades.^2^

Following liver transplantation, most of the pre‐existent portosystemic shunts (PSSs) regress and gradually become clinically irrelevant^.^ ^3^ However, in cases of the LDLT grafts with relatively smaller intrahepatic vascular bed, decompression of the portal system may not be as optimal as with comparatively larger deceased donor grafts. The PSSs may persist in 30–40% cases and can siphon off the portal flow, leading to reduced graft perfusion. Besides, such partial grafts from LDLT are subject to rapid regeneration, causing further increase in intrahepatic vascular resistance, also contributing to compromised portal flow^.^ ^4^, ^5^. Usually, the graft portal vein (PV) velocity is higher than that in a healthy adult and measures greater than 30 cm/s immediately post‐transplant. A low PV velocity, of less than 20 cm/s, can raise a suspicion of portal steal, and a velocity lesser than 10 cm/s has been considered unacceptable and requires immediate intervention.^6^ Depending on the amount of PSS flow, portal inflow may be diminished to various degrees. When there is a significant reduction of portal inflow resulting from PSS, it is referred to as “portal steal.”^5^, ^7^ Following LDLT, PSS may persist and could further jeopardize the portal inflow by diverting the portal blood. This could eventually lead to graft dysfunction and/or hepatic encephalopathy (HE) and rejection^.^ ^7^, ^8^ Moreover, in recipients with small‐for‐size (SFS), pediatric, and living donor grafts, iatrogenic portocaval shunts may be created to attenuate portal flow, thereby protecting the graft liver from the injuries associated with portal overperfusion and SFS syndrome (SFSS) (cholestasis, coagulopathy, and massive ascites with imminent sepsis). These surgical PSS can also subsequently lead to postoperative hyperammonemia and graft hypoperfusion crisis.^9^

Very little data have been published about endovascular techniques for managing portal steal in LDLT recipients. We herein present our experience with endovascular methods of occluding PSS in adult LDLT recipients, including retrograde transvenous obliteration of PSS (using vascular plugs, sclerosant, or coils) and occlusion of portocaval shunt by placement of covered graft in inferior vena cava (IVC).

## Methods

This retrospective study was conducted from March 2013 to March 2020 at a tertiary care hospital in North India. In this period of 7 years, over 2500 patients underwent LDLT at our Institute, of which five patients (0.2%) presented postoperatively with portal steal syndrome due to large PSS and were managed by endovascular interventional procedures. Written consent was obtained before the procedure. A review of patients' clinical and imaging records was conducted.

All five LDLT recipients (four males, age range 53–62 years, Table 1) presented to the Liver Transplant team with signs and symptoms suggestive of graft dysfunction, after a time period ranging from 3 months to 5 years after transplantation. The presenting symptoms included jaundice (*n* = 5), pruritus (*n* = 2), and altered sensorium with or without behavioral changes (*n* = 3). Liver function tests (LFTs) were deranged in all five patients with elevated serum bilirubin and liver enzymes (Table 2), with a worsening trend. Three patients thus were diagnosed with HE along with deranged liver enzymes. All five patients underwent initial conservative medical treatment for graft dysfunction and HE but were resistant and failed to show clinical improvement. Doppler evaluation in all the five patients was done and revealed reduced portal flow velocity, as detailed in Table 2. Contrast computed tomography (CECT) evaluations in these in all revealed prominent PSSs, with anatomy as detailed in Table 1.

**Table 1:** Patient characteristics and procedural details

S. no.	Age, sex	Etiology of pre‐transplant CLD	Year of liver transplantation	Type of PSS	Hepatic encephalopathy/clinical grade	Interval after transplant (months)^†^	Procedure	Details of device	Additional procedure
1.	62, M	Cryptogenic	2013	Meso‐caval	+/III	3	IVC stenting	Endurant II Stent graft system (Medtronic, Inc., Minneapolis, MN, USA)	PV stenting 2014
2.	49, M	NASH	2015	Splenogastrorenal + meso‐renal	+/II	24	PARTO (3Plugs) (Fig. 2)	10, 22, and 10 mm Amplatzer Vascular plug (Abbott, Chicago, IL, USA)	—
3.	57, M	Ethanol	2013	Surgical (Iatrogenic) PCS	+/III	60	IVC stenting (Fig. 1)	Zenith TX2 TAA Endovascular graft with Pro‐Form (Cook, USA)	—
4.	53, F	Hepatitis C	2017	Lieno‐renal	—	3	PARTO	20 mm, Amplatzer IV Vascular plug (Abbott, Chicago, IL, USA)	—
5.	53, M	Biliary Cirrhosis, EHPVO	2018	Lieno‐renal	—	6	PARTO + BRTO + Coiling of minor outflow veins	Amplatzer Vascular plug 14 mm and CODA Balloon Catheter‐40 mm (Cook Inc., Bloomington, IN, USA)	HV stenting‐2019, PTBD—2019

^†^ Denotes the interval period between liver transplantation and Endovascular shunt occlusion procedure.

BRTO, balloon‐occluded retrograde transvenous obliteration; CLD, chronic liver disease; HV, hepatic vein; IVC, inferior vena cava; PARTO, plug‐assisted retrograde transvenous obliteration; PV, portal vein.

**Table 2:** Pre‐ and post‐procedural Doppler findings

S. no.	Pre‐procedural Doppler evaluation				Post‐procedural Doppler evaluation	
	PV velocity (cm/s)	PV flow direction	HV flow	PV stenosis	PV velocity (cm/s)	PV flow direction
1	14.6	Hepatopetal	Triphasic	Non‐critical (7 mm, <30%)	25	Hepatopetal
2	8	Hepatopetal	Biphasic	No	28	Hepatopetal
3	10.5	Hepatopetal	Triphasic	No	35	Hepatopetal
4	5	Hepatofugal	Biphasic	No	8	Hepatopetal
5	13.5	Hepatopetal	Biphasic	No	23.5	Hepatopetal

HV, hepatic vein; PV, portal vein.

Graft liver biopsy was done for only one of these five patients who had a clinical suspicion of graft rejection (S. no. 4 in Table 1), and histopathological analysis confirmed acute graft rejection. Although decreased portal flow in this patient may have been partly attributable to rejection and was being treated by standard of care medical management and high dose of immunosuppressants, a multidisciplinary board discussed the feasibility and possible benefit of PSS occlusion in order to salvage the graft. Thus, despite a guarded prognosis, a PARTO procedure was performed in this patient, with intent to reverse the portal steal syndrome due to a large lieno‐renal shunt. Biopsy was not considered necessary on clinical evaluation by transplant hepatologists in remaining four patients.

Endovascular interventional techniques were used to counter the portal steal syndrome in all patients using jugular and/or femoral venous access. One of the five patients had an intraoperatively fashioned portocaval shunt (Table 1, S. no. 3) in anticipation of a SFSS due to a low graft‐to‐recipient weight ratio (0.69). The rest four had spontaneous PSS, which were not surgically ligated during transplant.

**Figure 1:**
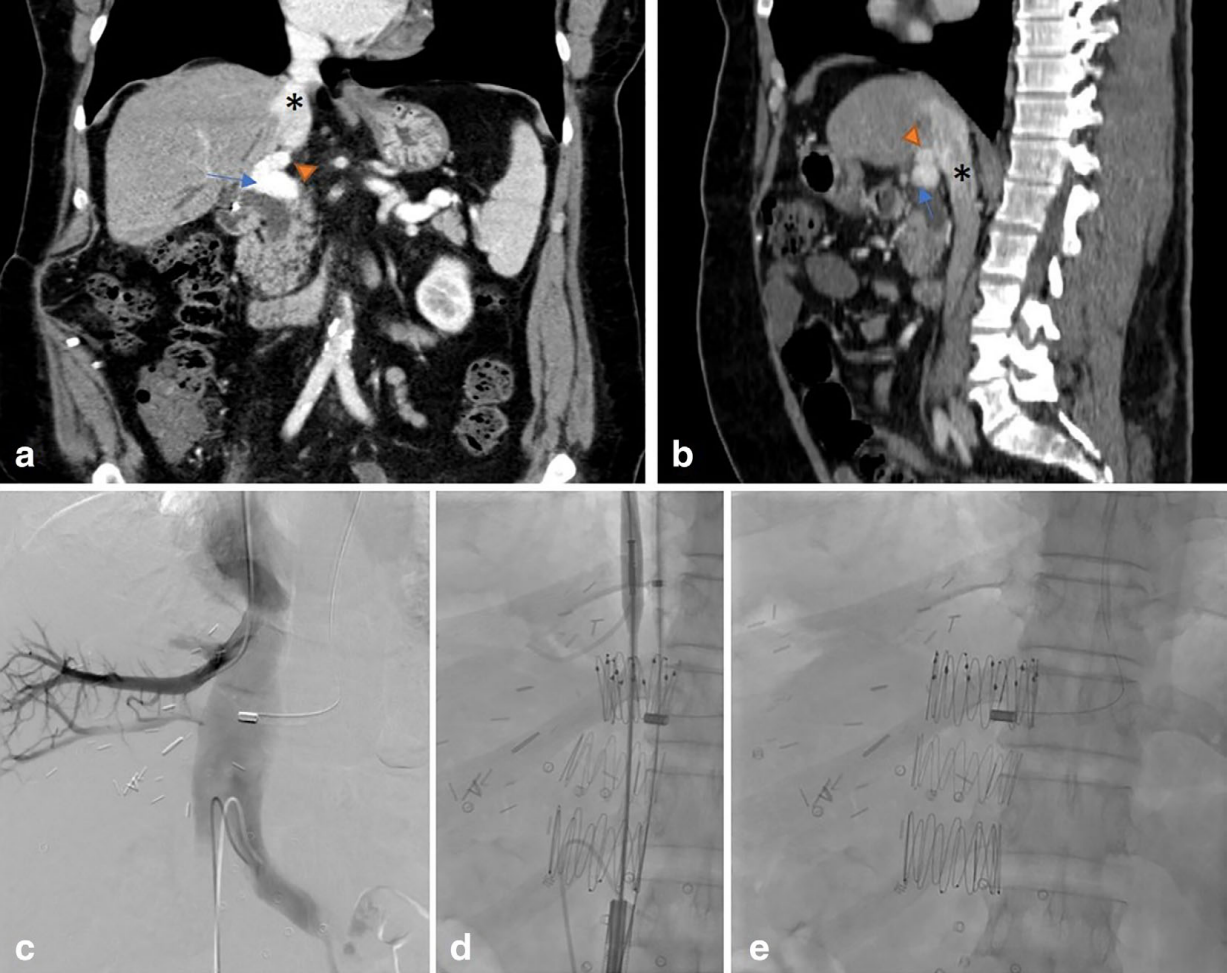
(a, b) Coronal and sagittal Maximum Intensity Projection CECT images showing a surgically created hemi‐portocaval shunt (orange triangle) between the IVC (*) and the right portal vein (blue arrow). Reformatted CT images were used to estimate the relation of the hepatic venous outflow and the Portocaval shunt outflow into the IVC to guide the IVC stent deployment. (c) Combined hepatic and IVC venogram was done to assess the landmarks for IVC stent placement and correlate it with pre‐defined CT measurements. (d, e) Metallic IVC stent graft deployed over a guidewire to obliterate the portocaval shunt. IVC, inferior vena cava.

**Figure 2:**
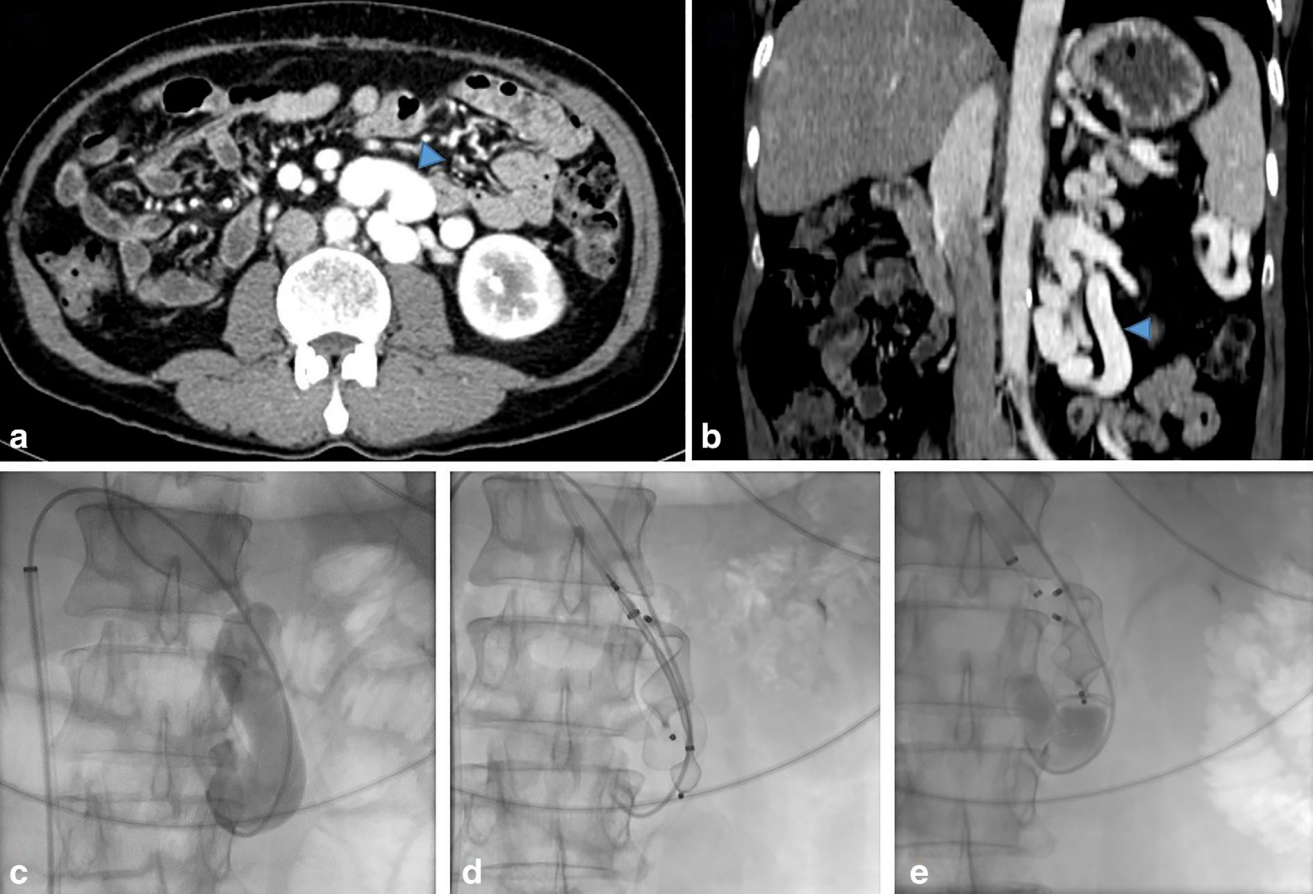
(a, b) Axial and coronal CECT images in a living donor liver transplantation recipient showing large portosystemic shunt with gastro‐splenorenal (blue triangle) and meso‐renal components. (c) Cannulation of shunt using C1 catheter with venogram demonstrating the tortuous collateral channel. (d) Vascular plugs used to occlude the shunt. (e) Post‐plug assisted retrograde transvenous obliteration venogram demonstrates no further retrograde flow into the shunt.

In two patients, an IVC stent graft (details in Table 1) was deployed at the level of the PSS, with intention to isolate the shunt and occlude the outflow of the large portocaval shunts (Fig. 1). The other three did not have shunts directly draining into the IVC, and hence retrograde transvenous obliteration of PSS was performed in these cases using vascular plug (plug‐assisted retrograde transvenous obliteration—PARTO, *n* = 3, Fig. 2) alone, or in combination with sclerosant (balloon‐occluded retrograde transvenous obliteration—BRTO, *n* = 1). In one patient (Table 1, S. no. 5), the diameter of the PSS at its outflow into the renal vein was very large, measuring more than 30 mm. Therefore, we decided to use a large 40 mm occlusive balloon for BRTO in this patient. Prior to occlusion of the dominant shunt using sclerosant, coils were used to embolize two thin outflowing collaterals in order to isolate the main shunt and thereby increase the dwell time of the sclerosant within the main shunt. A vascular plug was also used to obstruct the outflow efferent venous channel in order to increase the contact time of the sclerosant with the dominant shunt and achieve a stable cast formation. The sclerosant mixture (sodium tetradecyl sulfate [STS] foam, made using combination of agents with a 1:2:3 ratio of lipiodol:3% STS:air; volume—18 mL], was then used after balloon occlusion of the main shunt in this patient. Patient and procedural details are tabulated (Table 1).

### Data analysis and follow‐up

Technical success, complications, and long‐term (up to 1 year) clinical outcomes were assessed. We defined “technical success” as successful occlusion of portosystemic collaterals vessel with slowing/cessation of flow in the collateral and subsequent increase in hepato‐petal portal flow as documented on post‐procedural Doppler. Post‐procedural complications were considered major only if they necessitated additional intervention or resulted in adverse sequelae or death, and were categorized as minor if they did not pose any hindrance in management and did not require any further intervention. Clinical success was defined as improvement in liver function, clinical symptoms, clinical improvement in HE with or without improvement in blood ammonia levels in patients with HE, noted at 1‐year follow‐up visit (Tables 2 and 3). Patients were followed up for at least 1 year and all other interventions during the 1‐year follow‐up period were considered of consequence and recorded.

**Table 3:** Pre‐ and post‐procedural liver function test (LFT) at 1 year follow up

S. no.	LFT							
	Pre‐procedure				Post‐procedure (1 year follow up)			
	T. Bilirubin	SGOT	SGPT	ALP	T. Bilirubin	SGOT	SGPT	ALP
1	5.7	199	237	150	1.3	40	27	80
2	2.9	120	150	132	1.3	52	47	120
3	2.1	222	165	165	0.8	33	22	66
4	5.8	154	216	359	—	—	—	—
5	2.7	148	136	445	0.7	63	47	300

ALP, alkaline phosphatase; SGOT, serum glutamic‐oxaloacetic transaminase; SGPT, serum glutamic‐pyruvic transaminase.

## Results

### Technical success

All five patients were LDLT recipients, and were found to have large PSS on cross‐sectional imaging. Technical success was achieved in all five patients (100%) with an increase in portal flow velocity seen immediately after the procedure (Table 2).

### Clinical success

Overall clinical success was 80% (four out of five patients). Besides the deteriorating liver function due to portal steal, one of the five patients (Patient 4 in Table 2), was in ICU with multiple pre‐existent co‐morbidities and multi‐organ dysfunction. There was transient improvement in liver enzymes post‐procedurally. However, due to the overall poor medical condition, and despite a multidimensional overall attempt to salvage the graft, the patient died after 5 days due to multi‐organ failure.

The other four patients showed sustained improvement in LFTs with near normal reports at 1‐year follow up, as shown in Table 3.

Blood ammonia levels were tested in the three patients who had presented with HE along with liver function derangement. All three showed elevated levels of pre‐procedural blood ammonia levels. Two of these patients underwent post‐procedural blood ammonia monitoring, which showed significant improvement within 1 week of procedure as tabulated below (Table 4). There was no recurrence of HE in the follow‐up period in any of the three patients.

**Table 4:** Blood ammonia levels in patients with hepatic encephalopathy

S. no.	Pre‐procedural blood ammonia (μmol/L)	Post‐procedural blood ammonia (μmol/L)
1	69	N/A
2	106	28
3	100	9

Two of the surviving four patients underwent unrelated but additional IR procedures within the follow‐up period of 1 year. One of these patients (Table 1, S. no. 1) gradually developed portal vein stenosis (PVS) after the initial procedure for isolation of the meso‐caval shunt, and at a 6 month follow‐up showed critical stenosis with a diameter <50% of that of pre‐stenotic PV. Doppler showed an increased velocity of 100 cm/s at site of stenosis and therefore a successful PV stenting was done. The patient was followed up and showed good graft function at the 1 year follow‐up. Another patient (Table 1, S. no. 5), who underwent a combination of BRTO and PARTO for large lieno‐renal shunt occlusion, with resultant improvement in portal flow. Diagnosis of partial hepatic venous outflow obstruction (HVOO) was suspected only subsequently at follow up, on detecting turbulence at venous anastomotic site. A hepatic vein‐IVC pressure gradient was then measured and a raised value of 10 mmHg confirmed the presence of HVOO, which was then managed with balloon venoplasty and stenting. It is possible that hepatic venous obstruction became obvious on imaging once portal flow became optimal. This patient was, subsequently, noted to have dilation of posterior ductal system on a USG done at follow up 1 month after BRTO. MCRP confirmed the presence of an anastomotic biliary stricture. Therefore, a percutaneous transhepatic biliary drainage (internal‐external) was done with a 1‐year internal–external catheter exchange program, after which the stricture was remodeled and required no further intervention. There could have been an additional benefit of these procedures on the normalization of LFT's and liver function during the follow‐up examinations.

### Complications

One patient did not survive beyond fifth post‐procedure day due to multi‐organ dysfunction including graft dysfunction and subsequent cardiac arrest. However, no major or minor procedure‐related adverse effects were seen within the follow‐up period in any of the patients.

## Discussion

Liver cirrhosis can lead to portal hypertension due to increase in hepatic sinusoidal and intrahepatic vascular resistance. Subsequently, other pathophysiological factors, including splanchnic vasodilation and hyperdynamic circulatory syndrome play a role in formation of compensatory portosystemic collaterals, which may be seen in up to 40% of the cirrhotic population.^1^, ^10^

These preexistent PSSs are often seen to regress spontaneously^.^ ^3^. Such involution is more promptly seen with the use of cadaveric whole‐liver donor grafts, that is, in deceased donor liver transplantation (DDLT) as compared to partial liver/small size grafts in LDLT.^7^, ^10^ This occurs because of the response of portosystemic collaterals to the capacitance of the graft—DDLT grafts with a larger capacitance for portal flow, significantly reduce the intrahepatic vascular resistance, decompresses the portal system effectively, and thereby close down the PSS. In cases of the smaller LDLT grafts with decreased vascular bed, decompression of the portal system is not as optimal. Besides, these partial grafts are subject to rapid regeneration causing further increase in intrahepatic vascular resistance, also contributing to compromised portal flow^.^ ^4^ Following LDLT, therefore, PSS may persist and could jeopardize the portal inflow. Optimal hepato‐petal portal flow is essential for adequate regeneration of post‐LDLT partial grafts. The presence of large portosystemic collaterals or postoperative persistence of residual collaterals may lead to portal steal syndrome with subsequent graft dysfunction and/or HE.^7^

Diagnosis of portal steal requires a good radiological evaluation. Doppler US can accurately demonstrate the portal venous flow velocity and direction, while CT can help to provide anatomical details of the collateral vessels at different locations.^8^ The normal graft portal venous (PV) flow is hepato‐petal, showing a continuous spectral waveform with pulsatility and a mean velocity of more than 30 cm/s, resulting from a mismatch between the caliber of the smaller donor PV component (i.e. the right main branch) and the recipient's main PV component in right lobe grafts. Additionally, the recipient's large portal flow volume is directed to a partial instead of a whole native liver size.^6^

Detailed PubMed search did not provide an estimate percentage of LDLT cases that present with portal steal postoperatively; however, the incidence appears to be rare. In our experience with over 2500 LDLTs, we had five patients presenting postoperatively with portal steal syndrome, four with large spontaneous PSS, and one with a surgical portocaval shunt, amounting to approximately 0.2% cases.

At present, there is no accepted algorithm for managing spontaneous PSS before, during, or after liver transplantation, and evidence for efficacy of treatments remains limited. In a study by Ikegami et al.,^5^ retrospective analysis of 324 cases of adult‐to‐adult LDLT showed major PSS vessels (diameter >10 mm) in 130 recipients (40% of LDLTs), including a newly created hemi‐portocaval shunt. Of these 130 shunts, 116 were surgically ligated (89.2%). Reddy and Rela^10^ suggested intraoperative ligation of collaterals if they are large in size and present with low portal venous flow, with or without reduced portal pressures. In another study, Elshobary et al. reported a case of LDLT recipient with multiple collaterals and large lieno‐renal shunt. Intraoperative Doppler showed reduced portal flow likely due to siphoning of portal inflow. Therefore, ligation of the lieno‐renal collateral was done, following which there was prompt improvement of the PV flow on Doppler.^2^ Moon et al.^11^ reported two cases of portal steal after adult LDLT. They recommended prophylactic ligation of large portosystemic collaterals in adult LDLT, if its diameter is equal to or more than 10 mm, or less than 10 mm in diameter associated with PVS on the preoperative CT scan. In another study by Moon et al.,^12^ routine use of intraoperative portography was suggested as a technique to demonstrate steal and assess need for ligation of portosystemic collaterals.

Smaller spontaneous portosystemic collaterals may be left untouched either due to their innocuous appearance or due to technically difficult surgical access. However, there is a possibility that these initially small “natural” shunts eventually increase in size due to hemodynamic changes in portal flow, causing portal steal. Also, consistently high intrahepatic vascular resistance resulting from acute rejection, volume overload, congestion, or SFS graft can hinder spontaneous regression of the shunt.^8^ Additionally, in recipients of SFS liver grafts, iatrogenic portocaval shunts may be created surgically, facilitating partial diversion of portal flow into systemic circulation to avoid ill‐effects of portal venous overperfusion or persistent portal hypertension on the graft,^9^ as in one of our cases. These iatrogenic shunts may also subsequently cause portal steal, hampering graft function.

A few interventional radiological techniques have been described in the literature for obliterating PSS, causing portal steal and graft dysfunction in post‐transplant cases, including percutaneous embolization of shunt, transvenous obliteration of shunts using balloon occluded techniques (BRTO), plug‐ or coil‐assisted retrograde transvenous obliteration (CARTO/PARTO) of PSS, and transvenous endovascular closure of portocaval shunts using vascular stent grafts. However, these reports are few in number and most of them are in the form of individual case‐reports.

**Figure 3:**
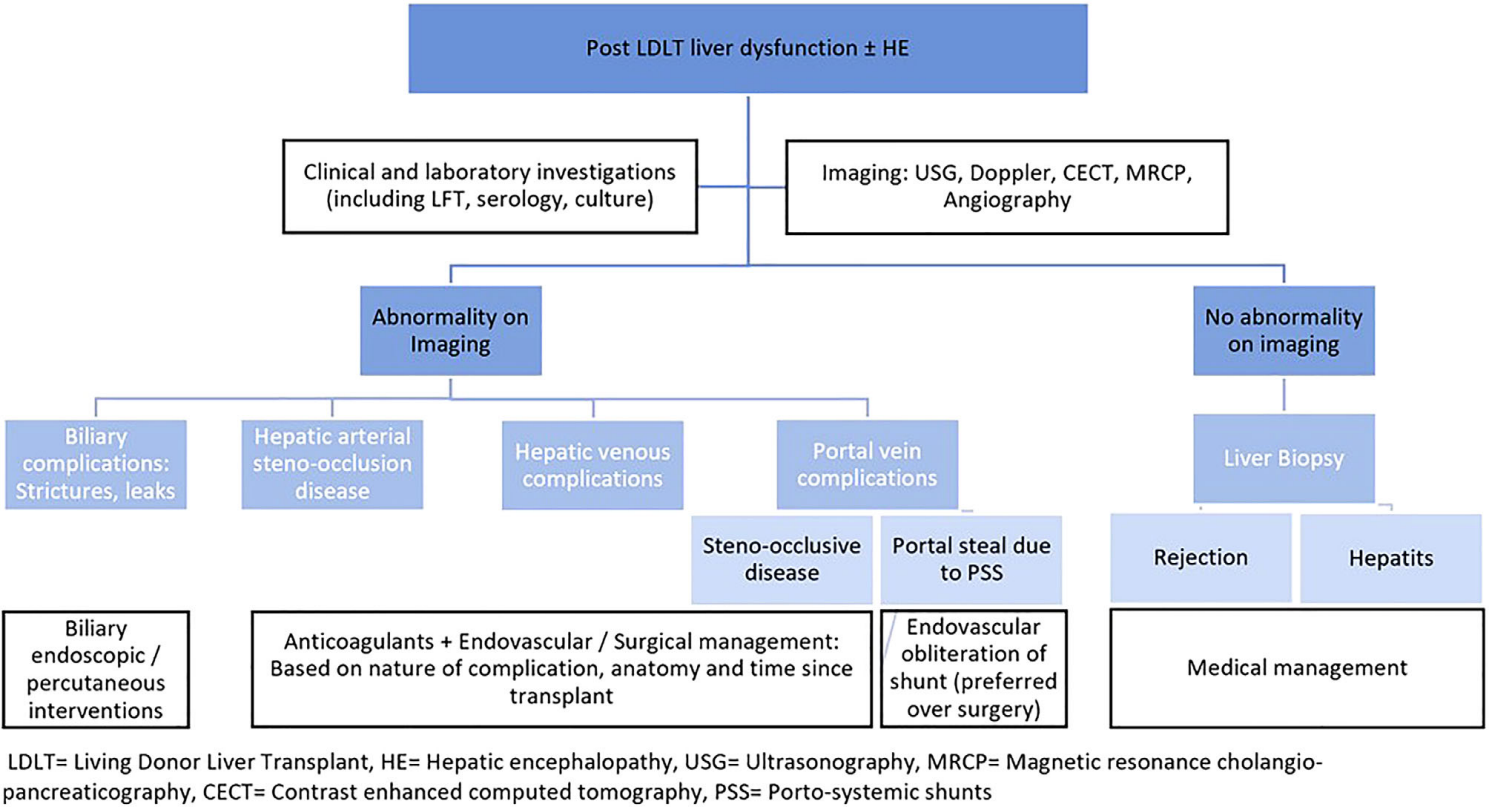
Suggested protocol for management of post‐living donor liver transplantation patients with deranged liver function test.

At our institute, the protocol for managing post‐LDLT patients with patent PSS and deranged LFT has been evolving with our experience over the past decade, as depicted in Figure 3. Transplant hepatologists clinically evaluate these patients for various causes of deranged liver function including graft rejection, infection, and biliary complications. Imaging further helps exclude vascular complications like hepatic arterial thrombosis, portal venous, and hepatic outflow obstruction, which are relatively rare, but treatable causes of deranged LFT in these patients. Interventional radiologists are involved if anatomic and hemodynamic abnormalities due to anastomotic vascular strictures, obstruction, or thrombosis are suspected. If present, management using minimally invasive endovascular techniques, like balloon angioplasty, stenting, etc., are preferred, in order to avoid re‐surgery as far as possible. In the absence of these vascular complications, portal steal phenomenon is suspected and investigated by cross‐sectional imaging and Doppler. If a large PSS is detected, endovascular techniques (like BRTO, PARTO, CARTO, and IVC stent grafts) are considered for occluding the shunts and increasing portal flow.

Botha et al.^13^ published a case report of a 48‐year‐old left lobe LDLT graft recipient who had a surgically devised hemi‐portocaval shunt (HPCS) in anticipation of a SFSS. Four months post‐transplant, the patient developed features of HE with elevated serum ammonia level, attributable to a steal phenomenon. After confirmation of the same on a portogram, a 26 × 39 mm aortic covered endograft was deployed in the IVC at the level of the HPCS, thus occluding its flow, with improved left PV flow at follow‐up imaging. Alhaizaey et al.^14^ reported a similar case in 2016, where in a 51‐year‐old, LDLT graft recipient, developed severe HE secondary to portal steal, and underwent IVC stent graft placement for isolation of the portocaval shunt, with post‐procedural clinical recovery within 2 weeks and obliteration of the PSS. Durack et al.^15^ used an Amplatzer muscular ventricular septal occluder device for closure of a small 13.9‐mm side‐to‐side PC shunt after left lobe liver transplantation.

In our series of five cases, we had two patients with portocaval shunts, one was an iatrogenic HPCS between the intrahepatic PV and IVC and the other was a spontaneous meso‐caval shunt. Both patients presented to us with HE, with elevated serum ammonia levels, and graft dysfunction. In both patients, we successfully deployed endovascular covered stent grafts (details in Table 1) at the level of the shunt in the IVC and there was definite clinical improvement seen in the follow‐up period. Also, in one patient, post‐procedural ammonia levels were measured and were found to have normalized.

BRTO and its variations like PARTO are established and effective procedures for treating HE due to a PSS related to cirrhosis of the liver. It has been extensively described in cirrhotic patients with PSSs and recurrent encephalopathy. Mukund et al.^16^ described seven patients with recurrent encephalopathy in whom BRTO was done for portosystemic collaterals. The authors reported 86% technical success with good clinical improvement maintained up to 3 months follow‐up period.

In another study from our Institute,^17^ five patients with cirrhosis underwent BRTO or PARTO for recurrent HE and the presence of lieno‐renal (*n* = 4) or meso‐caval shunts (*n* = 1). Sclerosant was used in two patients, endovascular occlusion plugs in two patients, and a combination of both in one patient. A significant reduction in serum ammonia was noted with no recurrence of encephalopathy in any of these patients.^17^ However, there are very few reports of these techniques in patients after liver transplantation. Baimakhanov et al.^18^ reported a case of a 52‐year‐old LDLT recipient with a large SMV–IVC PSS. Intraoperative shunt ligation was not done because there was sufficient portal flow into the graft after reperfusion. However, 1 year postoperatively, patient presented with HE. BRTO of the shunt was performed with effective management of encephalopathy and preservation of the graft function. In the present study, we performed PARTO in two patients, and combination of PARTO, Sclerosant, and Coils in one patient in order to obliterate the PSS in LDLT recipients. We noted 100% procedural technical success. Two of these three patients showed post‐procedural clinical improvement, while one patient was lost to follow up.

Kim et al.^8^ used percutaneous or direct portogram for the diagnosis and treatment of the portal flow steal. After localizing the steal by studying the flow direction on portogram, the route of the steal was obliterated either by embolization only or in conjunction with surgical ligation. Kim et al.^7^ also published a case of a 38‐year‐old LDLT recipient who presented with graft dysfunction secondary to portal steal via a large inferior‐mesenteric vein (IMV)—rectal varix. Direct transhepatic puncture of PV was done and portogram showed flow from the superior mesenteric vein (SMV) shunted mainly to IMV. Transcatheter embolization of IMV was done using coil and lipiodol and subsequent portogram showed no residual shunting and return of hepato‐petal flow in SMV.

HVOO can additionally contribute to portosystemic collaterals induced portal steal, especially in SFS grafts, as increased sinusoidal pressure may lead to persistence of these collaterals. Portal stenosis can also further worsen portal steal as the consequential resistance can lead to shunting of blood away from the graft. Therefore, in recipients presenting with portal steal, concurrent optimization of hepatic venous outflow and management of PVS are essential for restoring good graft function.^10^ PVS often presents as a late complication (>3 months) in adult LDLT recipients and usually occurs at the anastomotic site.^19^ Stenosis greater than 50% is considered hemodynamically significant. Although there are no consensus guidelines on ultrasound criteria for post‐LDLT PVS, usually a stenosis with a diameter <2.5 mm and blood flow aliasing with a flow velocity >80 cm/s at stenotic site, in the presence of signs of portal hypertension, are considered diagnostic with a sensitivity of 100% and a specificity of 84%^.^ ^20^

The diagnostic criteria for HVOO using Doppler ultrasound remain controversial. The normal spectrum of the hepatic vein is a triphasic waveform reflecting the cardiac cycle, but after transplantation the waveform can often be biphasic even without outflow obstruction. HVOO should be considered when a significant stenosis is revealed by the gray‐scale ultrasound or a high‐speed blood flow disorder appears at the stenosis. The ratio of stenotic to pre‐stenotic blood flow velocity is greater than 3–4:1 with a flat hepatic venous wave and slow or even reversed blood flow at the distal‐stenotic segment. A cutoff value of the pressure gradient between the IVC and the hepatic vein of 6 mmHg is also used to diagnose HVOO with a sensitivity of 86.7% and a specificity of 68%. Close monitoring and regular follow up with Doppler‐ultrasound is therefore extremely essential to pick up subtle signs of hepatic venous compromise.

In our study, we encountered one patient who developed critical anastomotic PVS, 6 months after PSS occlusion. Endovascular PV stenting was done within the 1‐year follow‐up period. Another patient had a right HVOO and an anastomotic biliary stricture contributing to the graft dysfunction and cholestasis, in addition to portal steal. Right hepatic vein stenting and internal–external percutaneous transhepatic biliary drainage were performed in this patient within 1 month of the BRTO/PARTO procedure. Both these patients showed good clinical improvement and normal LFT at the 1 year follow up. The limitations of our study include a small number of patients in the study group and a limited, 1‐year period of follow up.

We, therefore, present five cases where interventional radiological techniques were used to manage portal steal syndrome in post‐LDLT patients. Vascular plugs and stent grafts can be safely used to obliterate large PSSs in post‐LDLT patients to counter the portal steal, with clinical improvement in HE and liver function. Endovascular techniques with good technical results can, therefore, help in avoiding surgical exploration and ligation of such shunts.
